# An Accurate Machine-Learned Potential for Krypton under Extreme Conditions

**DOI:** 10.1021/acs.jpclett.4c03272

**Published:** 2025-02-04

**Authors:** Asuka J. Iwasaki, Marcin Kirsz, Ciprian G. Pruteanu, Graeme J. Ackland

**Affiliations:** SUPA, School of Physics and Astronomy and Centre for Science at Extreme Conditions, The University of Edinburgh, Edinburgh EH9 3FD, United Kingdom

## Abstract

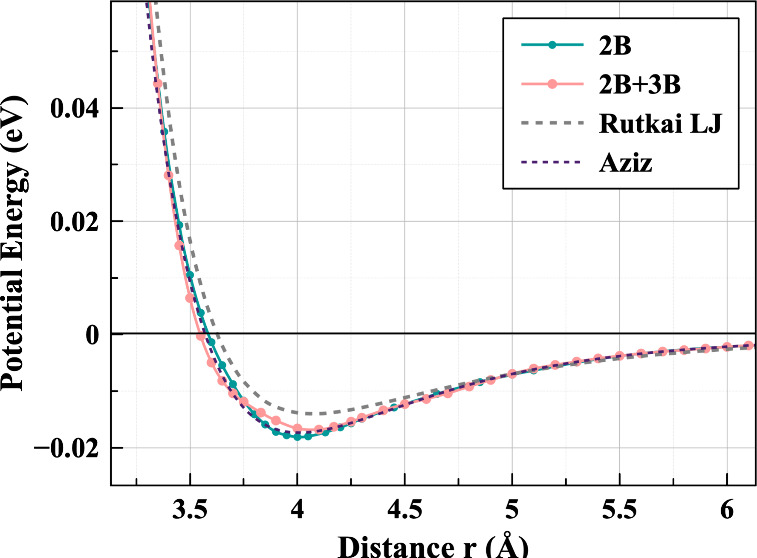

We have developed two machine-learned pair potentials for krypton based on CCSD(T) quantum chemical calculations on two and three atom clusters. Through extensive testing with molecular dynamics, we find both potentials give good agreement with the experimental equation of state, melting point, and neutron scattering data for the fluid. Compared with the most widely used Lennard-Jones model, our potentials produced similar results in low-pressure melting and equation of state. However, extending the regime to higher pressures of ≤30 GPa showed a remarkable divergence of the Lennard-Jones model from the experimental (solid) equation of state. Our potential showed extremely good agreement, despite having no solid phases in the training set.

Computational models of interacting particles can be used in the broad study of materials under different conditions. As a basis to describe the particle interactions in a molecular dynamics (MD) simulation which applies Newton’s laws of motion to the individual constituent particles of a material, an underlying potential energy function for the chemical species involved must be established. It is the derivative of this function which gives the interatomic forces required to command the MD integrator.

A number of methods have been developed to fit functional forms to empirically or computationally derived potential energy data.^1^ The choices of data and fitting method are based on the properties of the material and conditions of interest. For instance, it may be preferable to sacrifice some accuracy in higher order energy terms to reduce computational cost if they are justifiably negligible within the particular region of study. In other systems, it may be necessary to specify high accuracy descriptions of electronic structure where such details make a significant contribution, such as in the study of chemical properties.^2^

Machine learning methods provide promising new tools for the development of interatomic potentials.^3^ The new paradigm attempts to represent the potential energy surface (PES) of a material with a flexible mathematical function albeit lacking physical meaning. The key idea is to construct a map between local atomic environments and associate energy with them which in turn is obtained from higher order theory such as density functional theory (DFT) or coupled cluster calculations (CCSDT). The wide range of functional forms available allow to smoothly trade quantum mechanical accuracy against computational efficiency.

The Lennard-Jones (LJ) potential^4,5^ is a simple pair potential model which uses only two parameters - ε the dispersion energy (depth of potential well), and σ the van der Waals radius - the distance at which the potential between the two particles is zero.
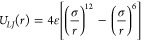
1

The *r*^–12^ term describes the repulsive behavior between the two particles and *r*^–6^ the attractive. As the LJ potential models an interacting pair of spherical particles, it has been considered reasonably representative of noble gases in particular.^6^ This is especially true for fluid states where many-body terms are widely thought of as having less significance than in solids and so a pairwise model provides a good approximation of the interactions, at least in the case of nonmetallic systems.^7^ Several generalizations and extensions of the potential have been proposed over the years, and shown to have improved accuracy with respect to reproducing certain thermodynamic properties of fluids of interest.^8−10^

However, in the particular testing of supercritical krypton by combined experimental and computational approaches, a number of shortcomings have been identified in the robustness of the LJ model.^11^ When shown in comparison to experimentally derived potentials (via Empirical Potential Structure Refinement - EPSR^12^), the repulsive *r*^–12^ region of the LJ is notably stronger than that of any potential that represents the data. As a consequence, the distance at which the potential is zero - the particle size - is greater in LJ. Similarly, the attractive *r*^–6^ term increases more rapidly out of the potential minimum, leading to weaker attractive interactions between particles than needed to accurately represent the experimental diffraction data.^11^

We here produce two interatomic potentials for krypton trained on CCSD(T) data using the machine-learning (ML) software Tadah!.^13,14^ One potential is trained only on two-body data and the other includes additive three-body terms. We assess the performance of each fitted potential through structural measurements of radial distribution functions and equations of state, and thermodynamically by determination of melting curves. We compare and contrast these results with equivalent ones deploying the Lennard-Jones potential and with experimental data in order to evaluate the effect of three-body energy corrections and the quality of potentials developed by this method in relation to other models. We find that while the LJ model offers a relatively satisfactory agreement with experimental findings at low pressures (MPa range) and densities, especially for fluid phases, our models are significantly more robust in their transferrability to higher pressures (GPa range) as well as solid phases. One this latter issue, we find our three-body trained potential to show excellent agreement with most recent experimental determinations of the equation of state for solid krypton up to 35 GPa.^15^

## Methods

Potential energy data from Jäger et al.^16^ were used as the training data set for the machine learning process. This source provides 36 data points for potential energy between pairs of Kr atoms as a function of separation distance *V*(*r*), from 2.2 Å up to a distance of 15 Å. This available data range acts as a limit of validity of any trained potential, which will lose accuracy at pressures/densities for which the atom–atom separation in the material will be ∼2.2 Å. The data were generated through CCSD(T) (coupled cluster single–double and perturbative triple); a computational chemistry technique which can produce high-accuracy energy data for medium-sized systems.^17^ Jäger et al.^16^ used a specifically developed aV6Z basis set that is included in their study’s Supporting Information.

Also provided in the data source^16^ are three-body energy corrections to the pair potential obtained by fitting the energy for 11 equilateral triangle configurations of three Kr atoms, for atom–atom separations between 2.5 and 6 Å. These corrections Δ*V*_3_ are to be added to the pair energies *V*_2_ as
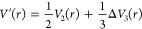
2to give the energy per atom *V*′. Their addition results in a slight shifting of the potential well to a lower *r*. Since the CCSD(T) three-body energies are given only for a fixed trimer configuration rather than as a function of angle, they are implemented as an additional pairwise term. The fitting process for this modified training set is the same as for the case employing only two-body data: Tadah! is used to fit a pair potential as before with the difference being the number of fitting functions used and their size and spacing.

As the three-body corrections^16^ are given only up to a maximum distance of 6 Å, uncorrected two-body energies were used to make up the full training data set to 15 Å. This is based on the assumption that the three-body corrections will be negligibly small at higher separation distances, such that the two-body terms should adequately describe the potential at this range. This is supported by the relative magnitude of the 3-body correction and the 2-body terms in the range where they are available, with the former representing at most 5% of the energy and decreasing with increasing distance (Figure 2). Some of the three-body correction data are from CCSD(T) calculations for interstitial distances where there is no equivalent two-body potential value. For these points, the two-body data were linearly interpolated to obtain a value for *V*_2_.

Tadah! was used to produce two pair potentials; one including three-body corrections and one without. For higher pressure systems, many-body interactions begin to have a more significant contribution to the energy of the system so we anticipate the inclusion of three-body terms to result in a better performing potential in this regime. The structure and thermodynamic behavior of the bulk material based on each potential was then assessed using LAMMPS^18^ molecular dynamics simulations. The resulting potentials are available in the native Tadah! format and as a tabulated LAMMPS format, compatible with standard LAMMPS, as it is a two-body interatomic potential. The Tadah! format requires a LAMMPS plugin. They can be downloaded from the Tadah! Web site.^13^

Tadah! is our machine learning software specifically designed for the development of custom-made machine learning interatomic potentials.^19^ The software is written in C++ with an easy to use command line interface. The trained models can be rapidly deployed to LAMMPS via a provided plugin. The software is open source and publicly available from https://tadah.readthedocs.io/. Tadah! provides a wide range of flexible mathematical fitting functions (descriptors) suitable for a wide range of problems as well as linear and nonlinear (kernels) machine learning models.

The accurate description of the potential energy surface is essential for the study of Kr with MD simulations. In our model, the total energy of the system, *E*_*total*_, is obtained by accumulating all local atomic energies, *E*_*i*_
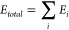
3where *E*_*i*_ is computed by summing over all pairwise interactions within the cutoff distance.

To obtain *E*_*i*_ we used a linear kernel with blip functions (eq 5) as the descriptors such that
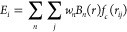
4where *B*_*n*_ is a blip function, as defined in eq 5, *f*_*c*_ is a cutoff function (eq 6) and *w*_*n*_ is an optimized regression coefficient; *r*_*ij*_ is the distance between *i* and *j* atoms and *r* its scaled equivalent such that *r* = η(*r*_*ij*_ – *r*_*s*_). Here *r*_*s*_ and η are hyperparameters which position blips on the grid and control their width, respectively.
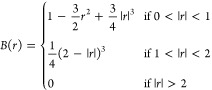
5

**Figure 1:**
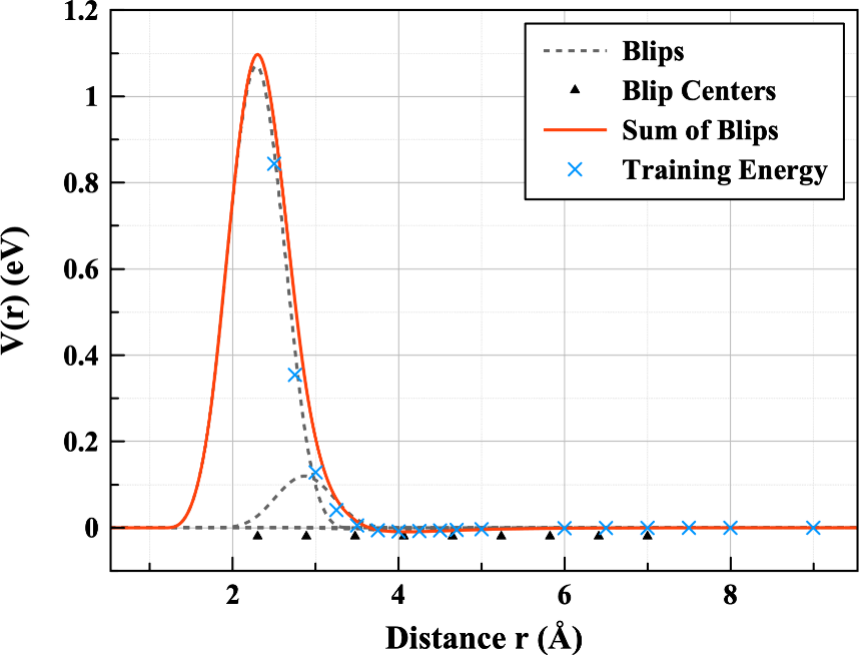
ML fit to the 2B+3B CCSD(T) data, showcasing how the addition of blip functions reproduces the potential energy curve.

The number of blips, their positions, and widths are hyperparameters which are optimized manually. The blip arrangement is illustrated in Figure 1. A cutoff function and distance are also specified in the training configuration file to ensure the potential smoothly goes to zero. Here we used the cosine function below with the cutoff distance *r*_*c*_ at 9 Å:
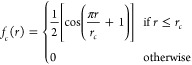
6

The resulting potential is a linear combination of blips, where the weighting of each blip is calculated by the Tadah! fitting process. Figure 1 shows how the sum of the blip functions predicts energies from the training data. It can be noted that at very short-range, in the absence of fitting data, and by design of the blips, the potential returns unphysically to *V*(*r*) = 0. In this work, the height of the barrier between 2 and 3 Å is sufficient to prevent any atoms accessing this region in our MD simulations. This could be easily fixed by adding a short-range repulsion,^20,21^ but since it proves unnecessary we chose not to do this.

To avoid overfitting, the number of blips should be less than the number of training data points. The widths should be adjusted such that there is sufficient overlap between neighboring blips to ensure a smoothly fit curve. Predicted energy points can be found for any distance chosen by the user. In general, a trial and error approach was taken to arrive upon an appropriate combination of the hyperparameters.

**Figure 2:**
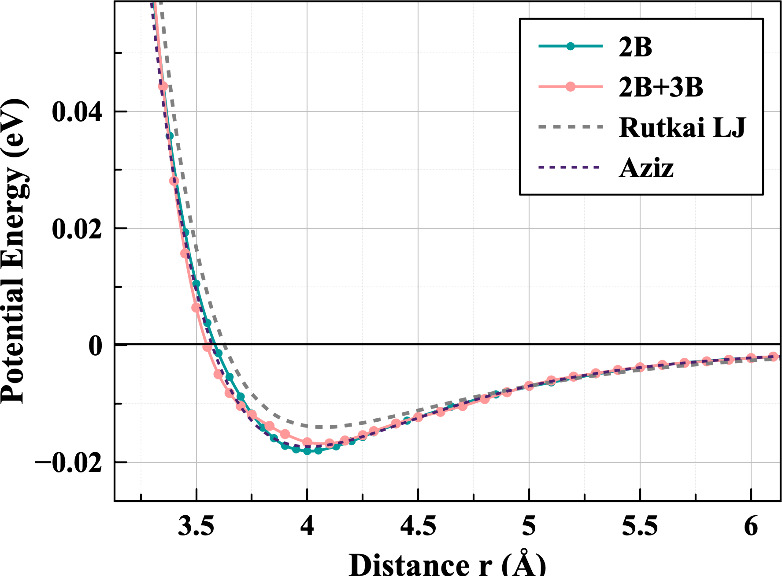
Comparison of the three potential energy curves employed in this study: the Lennard-Jones model of Rutkai et al.^6^ and our 2B and 2B+3B machine-learned potentials, along with the Aziz potential.^22^

Figure 2 shows our two resulting potentials compared with the Lennard-Jones model as fitted by Rutkai et al.^6^ The inclusion of three-body corrections broadens the pair-potential well. This figure also illustrates clearly the difference made by the inclusion of 3-body corrections throughout the range of the interaction potential, and supports the assumption that they are negligibly small at higher separations. It also shows that the widely used Aziz potential^22^ is remarkably similar to our 2B potential. The Lennard-Jones potential has a much stronger repulsive region than either of the ML fitted potentials. However, the attractive regions seem to agree much more closely than when compared with potentials derived from neutron scattering data using EPSR.^11^ Our fitted potentials show weaker hard-core repulsion effects than than LJ, so differences might become more apparent as pressure is increased in our MD simulations.

The equation of state and the structure of a simple liquid are fairly straightforward properties to compute. The melting curve is a much more stringent test. We calculate it here using two different standard MD-based methods, namely the Z method and phase coexistence. A more detailed, in-depth overview of current methodologies can be found in Karavaev et al.^23^ and in Zou et al.^24^ It is worth mentioning that previous studies employing first-principles calculations and many-body potentials derived from them have shown krypton to be particularly liable to superheating effects and to show a strong sensitivity to 3-body corrections.^25^

**Figure 3:**
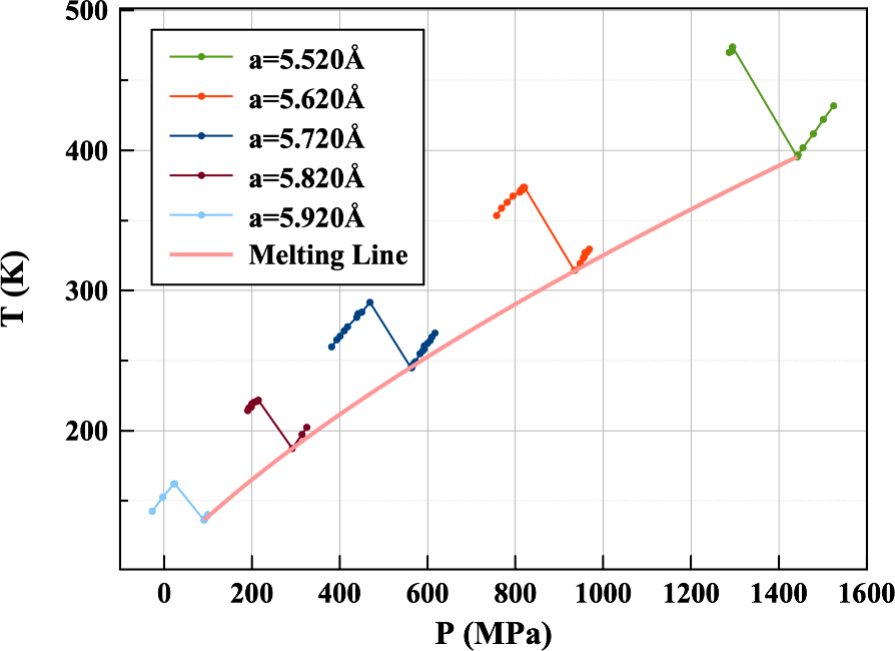
Melting line for two-body fitted Krypton potential starting from FCC by Z method. Different NVE volumes are set by adjusting the FCC lattice parameter *a*.

The Z method is a technique by which melting curves can be determined from MD simulations.^26^ A series of NVE (microcanonical) simulations are run, each with a different initial temperature which dictates the particle velocities and therefore the energy of the system. Figure 3 shows how individual NVE simulations of the same volume and different energies form Z shaped isochores on a T-P phase diagram. The point at which the isochore drops in temperature is taken as one point on the melting curve.

The NVE simulation corresponding to this point is the lowest energy system of the chosen volume which melts. It is the temperature and pressure of the resulting liquid of this melted NVE system which are used to locate the melting point, in other words - the lowest temperature liquid which can be found through melting on this isochore.

The Z method works by superheating the NVE solid until melting occurs. As a system melts under NVE conditions, the temperature drops due to the latent heat of fusion and the pressure increases to accommodate the volume remaining constant across the phase change. The Z method has been demonstrated to produce melting curves with good accuracy for various materials including metals,^27^ covalent compounds,^28^ and clay minerals such as kaolinite.^29^

**Figure 4:**
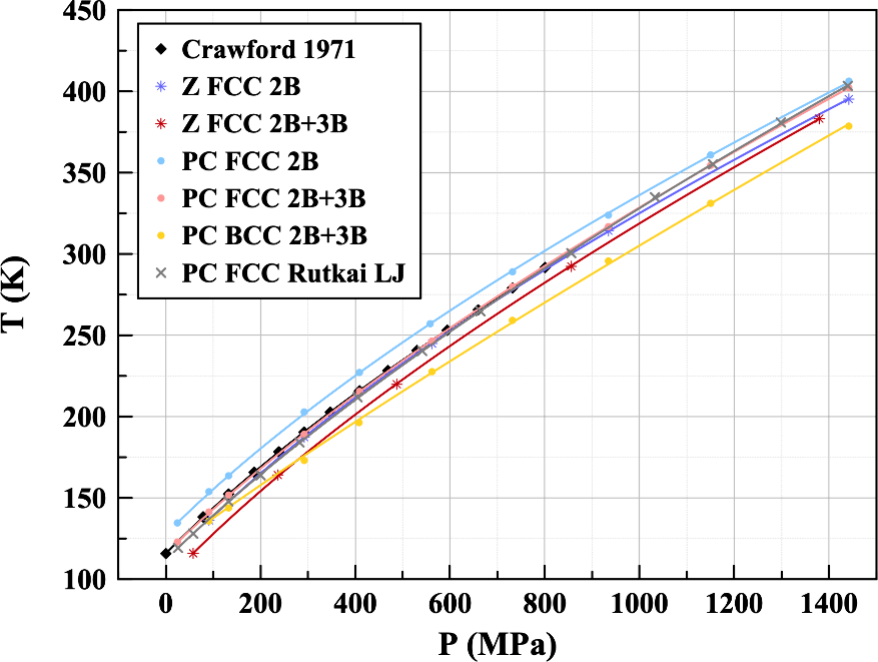
Melting lines produced by the Z method and phase coexistence simulations with 2B and 2B+3B potentials. An alternative crystal structure, BCC, is also shown for a lattice stability comparison as well as the experimental and Lennard-Jones results.

All simulations were initialized with FCC structure, the expected stable solid structure for this region.^30^ Z method melting lines were produced for both potentials (Figure 4). The melting points have been fitted with the Simon-Glatzel equation.^31^ An experimental melting curve^32^ is also shown for comparison for pressures up to 1 GPa. The melting curve of krypton has also been reported at significantly higher pressures than considered here,^33^ however the data points are quite sparse and have been found to be inconsistent with recent *ab initio* Monte Carlo calculations,^34^ so we opted not to attempt a direct comparison to these.

Both potentials seem to produce similar melting lines close to the experimental result with the 2B giving a slightly higher melting temperature. The inclusion of three-body corrections lowers the melting curve which follows from the weaker London dispersion forces resulting from further-body terms requiring less energy to melt the solid. It is unclear, however, why this melting curve should be in poorer agreement with experimental findings.

In order to provide some confirmation of the Z method results, the solid–liquid phase coexistence method was also used to generate these same melt curves.

Solid–liquid phase coexistence (PC) is another method of determining melting temperatures with molecular dynamics.^35,36^ A cuboid simulation box of atoms is designed with half of the box in the liquid state and the other half in the solid state. As the system equilibrates in the isobaric-isenthalpic (NPH) ensemble, the interface between the two phases may move as the solid melts or the liquid crystallizes. When the interface has stopped moving, the coexistence temperature - melting temperature - has been reached. The temperature and pressure of this state make up one point on the melting line. It is worth noting that despite maintaining both liquid and solid states within the simulation, this method is distinct from “interface pinning”,^24,37^ in that no bias potential is employed.

**Figure 5:**
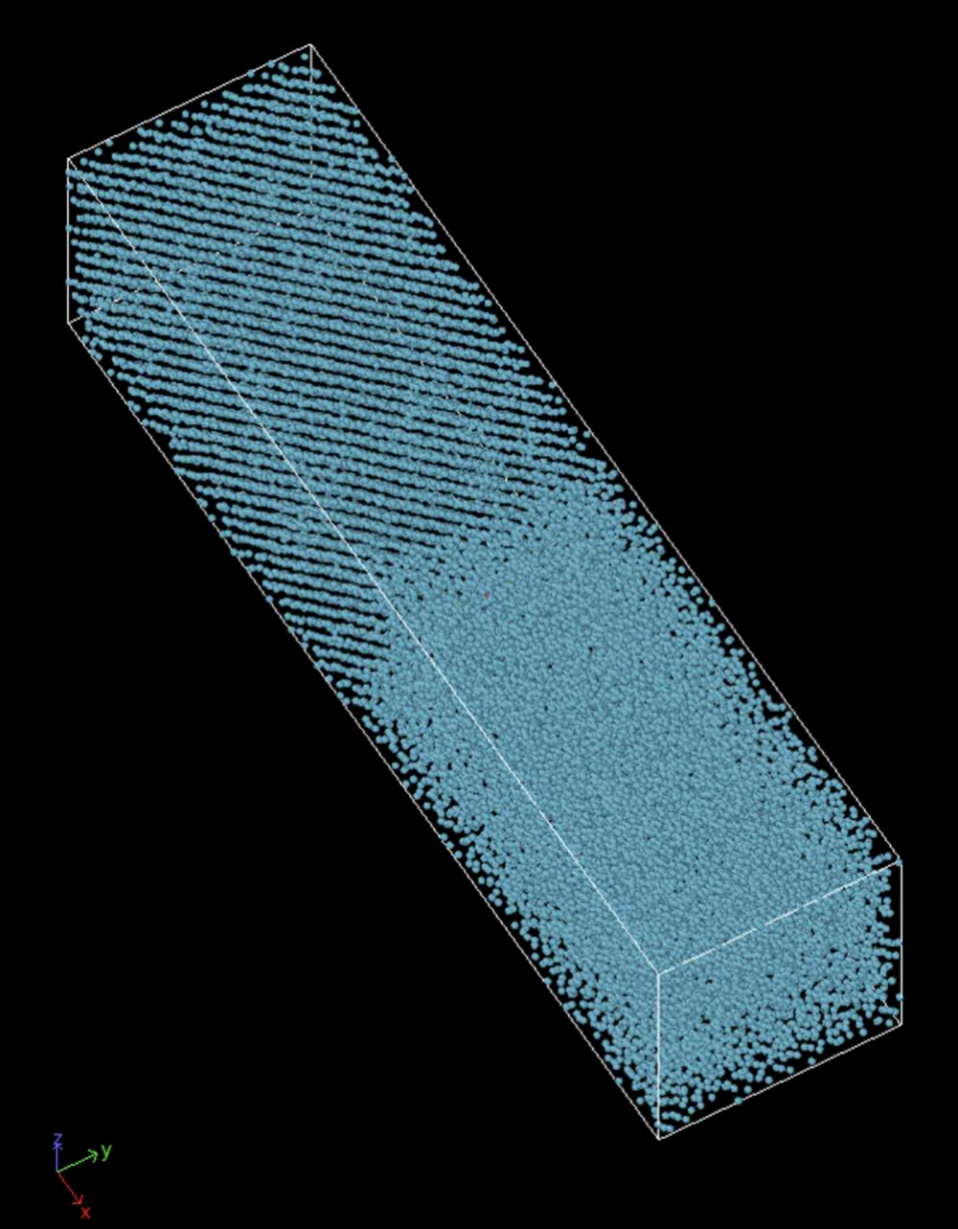
LAMMPS solid–liquid phase coexistence box with periodic boundary conditions.

We constructed a phase coexistence simulation in LAMMPS using a box populated with 28800 Kr atoms in FCC configuration (Figure 5). The entire system is first equilibrated to an estimated melting pressure and temperature by running in the isobaric–isothermal (NPT) for 250 ps. Then, the atoms in one-half of the box are heated to a higher temperature via another 250 ps NPT run. The heating temperature is approximately three times the equilibration temperature in order to ensure a liquid state is reached. These atoms are then cooled back down to the equilibration temperature via a third 250 ps NPT run. Finally, the entire system is run in the NPH ensemble. The NPH must run long enough for the system to demonstrate a stable solid–liquid coexistence state. The initial equilibration variables may be adjusted until such a state is found.

Phase coexistence produced melting curves for our two potentials in close agreement with experimental and Z method results (Figure 4). A similar difference between the 2B and 2B+3B potentials appears as it did in the Z method. However, the PC method seems to produce melt lines which are systematically slightly higher in temperature than the Z method does.

In order to establish the stability of the FCC structure up to melting, solid–liquid phase coexistence simulations starting in BCC configuration were also performed. BCC phase coexistence followed the same procedure with the simulation box now populated with 14400 atoms in a BCC lattice. The resulting curve yielded lower melting temperatures than FCC, suggesting a higher Gibbs free energy and therefore a less stable solid structure for this region. Hence the model predicts that FCC remains stable up to the melt line, consistent with experiment.^38^

Finally, a melting line for the Lennard-Jones model was found via phase coexistence. The result also shows very close agreement with experimental data and the 2B+3B phase coexistence line. This result seems to suggest that two visibly differently shaped potentials (LJ and 2B+3B (Figure 2)) can, through the same method, produce near-identical melting behavior in this region. Low-pressure melting (up to 1.5 GPa) appears to be within the capability of the LJ potential to accurately model krypton. This is in good agreement with previous studies by Mastny and de Pablo,^39^ who investigated krypton within the Lennard-Jones potential at lower pressures and temperatures, and highlighted the effect of finite cutoff on the reported melting temperature.

NVT (canonical ensemble) MD simulations allow us to test various properties of the material in thermal equilibrium. We will look in particular at the isothermal equation of state and radial distribution function (g(r)). We have used for comparison a set of data^11^ derived using EPSR - a technique by which a potential energy function and structural model can be derived from neutron scattering data.^12^ We compare *g*(*r*) and EoS measurements of our two fitted potentials with Lennard-Jones and EPSR in order to identify the structural consequences of the different potential models.

The LAMMPS simulation box was initialized with 2048 atoms, FCC structure, and lattice parameter *a* = 5.720 Å. Using *T* = 310*K* and the seven different pressures studied in the neutron total scattering experiment, an equilibrium volume for the chosen temperature and pressure was first obtained by running a 40 ps NPT simulation. Then, using the PV conditions reached at the end of the NPT run, the system was run for 1 ns in the NVT ensemble and the resulting pressures, densities, and g(r) were analyzed.

The radial distribution function g(r) is a measure of the probability of finding another particle as a function of distance from the center of a particle. The particle trajectories from the NVT simulations were used to generate a g(r) for each tested pressure using the Visual Molecular Dynamics (VMD) software.^40^

**Figure 6:**
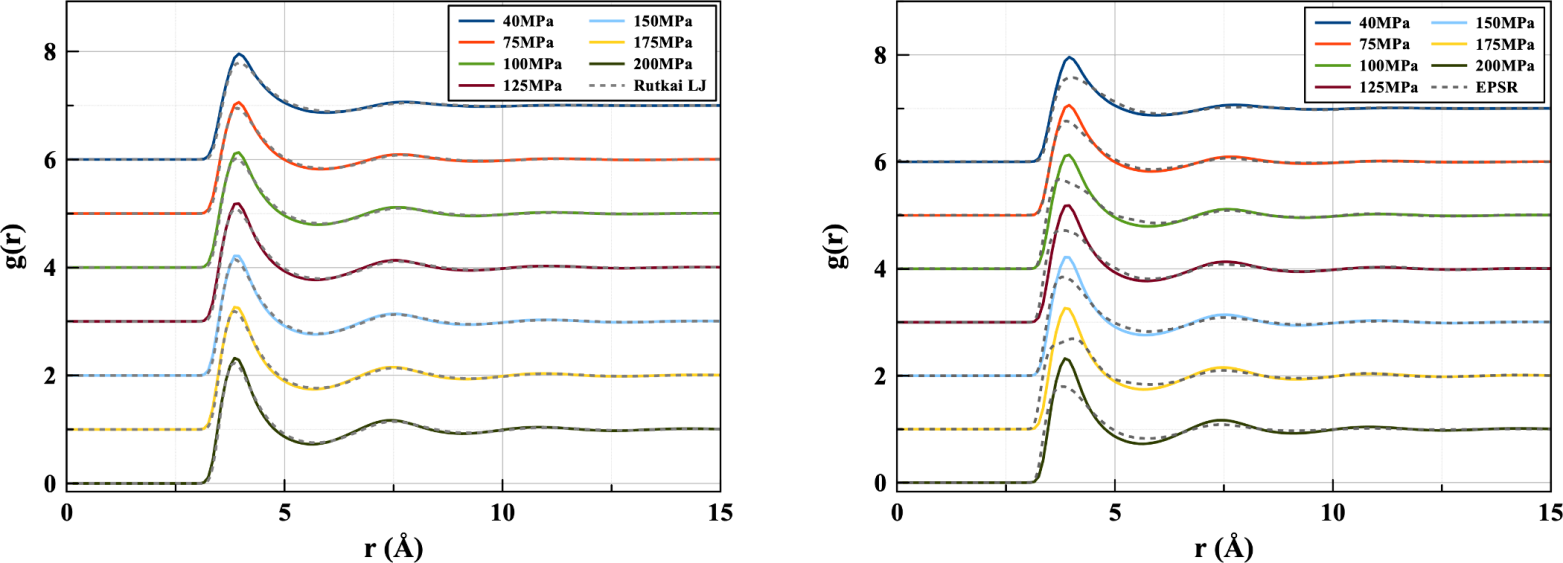
Radial distribution functions produced by the two-body Tadah! potential in comparison to the Lennard-Jones model (left) and Neutron scattering (right) at 310 K.

It was reported in the neutron scattering study employing EPSR^11^ that the LJ model deviates from the experimental result in the sharpness of the g(r) peak. This implies that the narrower well of the LJ potential leads to an overstructured fluid phase. A similar result can be seen when comparing the 2B Tadah! potential with LJ and experimental results (Figure 6). The two pair potentials -2B and LJ - show a similar overstructuring tendency which seems to be slightly stronger in the 2B potential at lower pressure. With increasing pressure, the peaks draw closer.

**Figure 7:**
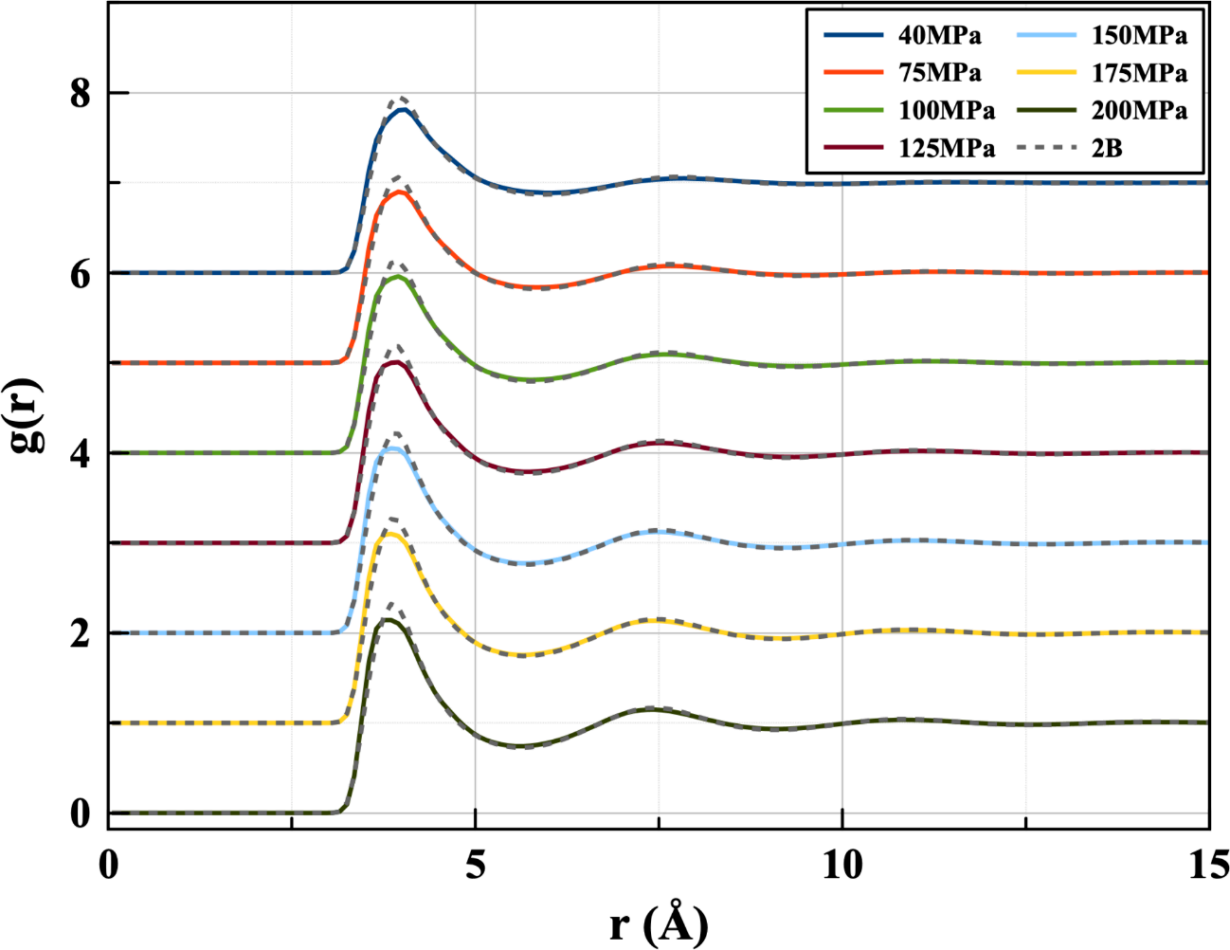
Pair distribution functions comparing the ML two-body potential and three-body corrected potential. Solid color lines show three-body *g*(*r*)’s for different pressures at 310 K. Gray dotted lines show the corresponding two-body results.

**Figure 8:**
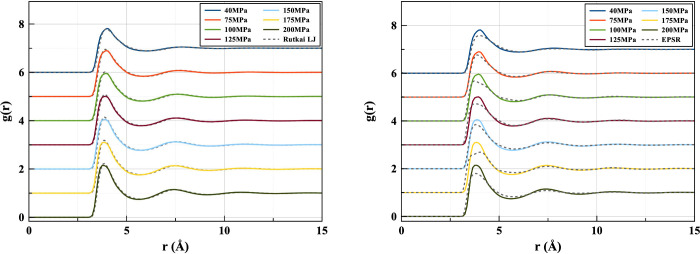
Pair distribution functions produced by the ML fitted potential including three-body corrections in comparison to the Lennard-Jones model (left) and EPSR-derived (right) ones at 310 K.

In Figure 7 it can be seen that the 2B+3B potential shows this overstructuring propensity to a lesser extent than the 2B. There is a much closer resemblance between the LJ and 2B+3B *g*(*r*) (Figure 8), possibly as a result of the breadth of the potential wells being more alike. Though the lowest pressure (40 MPa) g(r)’s seem to match almost exactly, a slight deviation begins to appear as the pressure is increased.

There are many proposed functional forms for equations of state (EoS), depending on the different assumptions made about the interatomic interactions within the material. They are typically developed by introducing constants to adjust a starting form to better fit experimental data for different materials and conditions. A numerical isothermal equation of state can also be derived directly from NVT simulations. This gives the relation between density and pressure for a chosen temperature, from which one can also determine the compressibility of the material.

**Figure 9:**
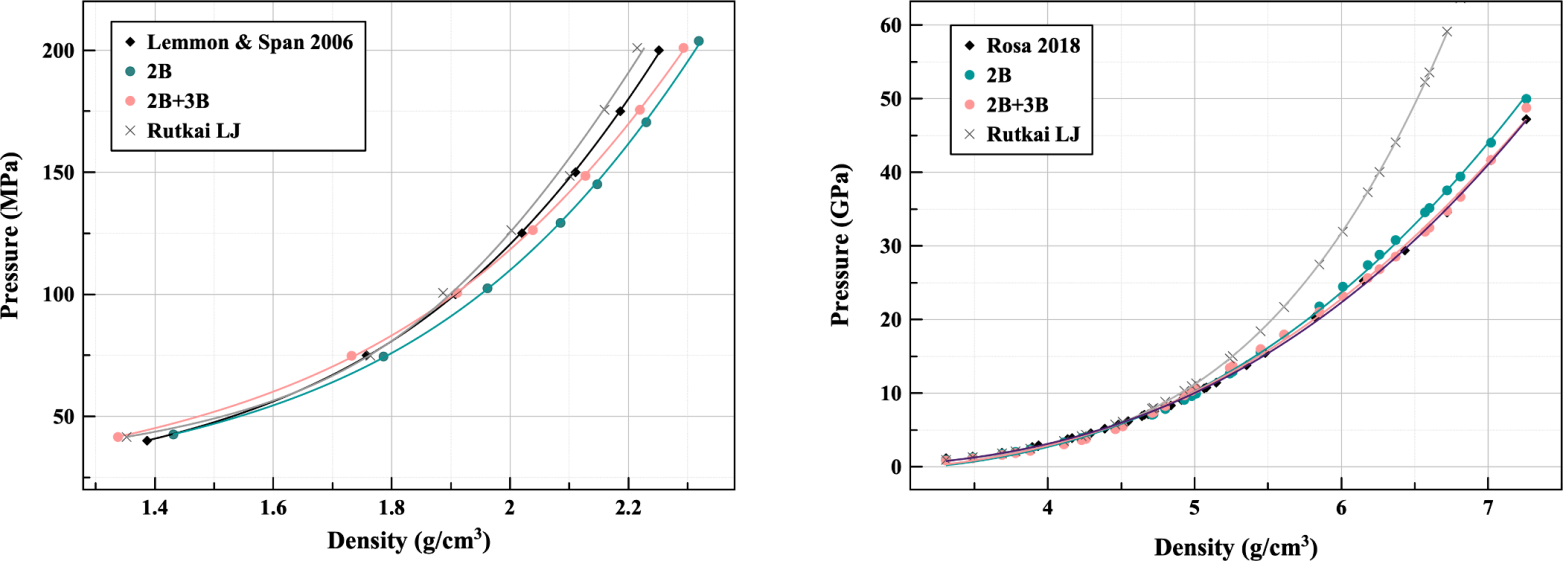
Comparison of the isothermal EoS of krypton from experimental, Lennard-Jones, and two Tadah! fitted potentials: (left) supercritical fluid and (right) FCC solid.

The isothermal EoS produced from LAMMPS NVT simulations (Figure 9 (left)) for the different models is compared to the universal equation of state for krypton by Lemmon and Span.^41^ The different potential models show a degree of overlap in the lower pressure region of the supercritical fluid EoS which begin to separate as pressure increases. In order to further investigate the differences which seem to emerge with increased pressure in the fluid EoS and related pair distribution functions, the determination of the EoS was extended to higher pressures - into the solid phase of krypton. Shown in Figure 9 (right) are the solid FCC EoS of our Tadah! potentials compared with the most recent experimental results of Rosa et al.^15^ and LJ. In this regime, the effects of the repulsive component of the potentials become more pronounced. Though at lower pressure all models seem to produce similar curves, the Lennard-Jones deviates significantly from the experimental result as pressure is increased. To a lesser extent, the 2B Tadah! potential similarly begins to deviate when the pressure is increased further. The 2B+3B Tadah! potential stays extremely close to the experimental results throughout the entire tested range. It would appear from this that the inclusion of three-body data does well to correct the incomplete character of the pair potential and offers a stark improvement over the Lennard-Jones model when applied to a broad pressure range, increasing the range of validity and tranferrability of the underlying potential.

In this study, we have produced two computationally efficient machine-learned interatomic potentials for krypton. The potentials were trained on data available from high accuracy quantum chemical CCSD(T) calculations previously reported by Jager et al.^16^ The first potential was trained exclusively on two-body energy contributions, while the second included minimal 3-body energy corrections arising from equilateral triangle configurations. The resulting potentials are composed as a summation of blip functions reproducing the potential energy curve between two atoms at varying separation.

We find that in the low-pressure regime (below 1.5 GPa) the two potentials produce similar results, in good agreement with the experimentally measured melting curve of krypton, stable solid phase and fluid and solid equations of state. Moreover, the results are in equally good agreement with the Lennard-Jones model of Rutkai et al.^6^ The differences between the potentials appear subtle, and manifest in the tendency of LJ and our two-body potential to overstructure the supercritical fluid when compared to experimentally derived structures, a tendency that while still present is nevertheless ameliorated in our three-body trained ML potential.

A very different picture is readily revealed once the range of pressure is extended into the multi-GPa region. We compared the performance of the 3 potentials (the 2 ML ones produced in this study and Lennard-Jones) with the most recent experimental studies on krypton by Rosa et al., up to 35 GPa. The LJ potential deviates heavily (beginning ∼10 GPa) from the experimental equation of state at ambient temperature, resulting in a 8 GPa upshift from the experiment at similar high densities (∼24 GPa in experiment vs ∼32 GPa in the simulation at a given density). This deviation appears strongly density-dependent, rendering the LJ parametrization of limited practical use for high pressure studies.

In contrast, our 2B energy trained potential follows more closely the experimental data up to higher pressures, but does still show a pressure upshift (starting ∼23 GPa), leading to a 2 GPa upshift at certain high densities (28 GPa experiment vs 30 GPa in simulation). An excellent agreement with the experimental reports throughout the entire pressure range is achieved by the 3B energy corrected ML potential. The main difference between the 3B and the 2B/LJ potentials is the reduced harshness of the repulsive part of the interaction, where 3B energy correction soften this, manifesting subtly in the structure of the supercritical fluid at low pressures (below 0.3 GPa), but becoming readily noticeable for the solid at high pressure (above 10 GPa).

Our ML potential trained on 3-body corrected energies from CCSD(T) calculations is accurate from ambient conditions up to 35 GPa, insofar as the equation of state, melting curve, stability of solid phases and structure of solid and supercritical fluid krypton are concerned. In contrast, the best-fit Lennard-Jones form gives overly strong repulsion at short-range. Surprisingly, the necessary physics for condensed-phase calculation can be deduced from calculations on two and three atom clusters, without any need to include condensed phase data in the training set.

## Supporting Information Available

Transparent Peer Review report available (PDF)
