# Supplementary material for: An Accurate Machine-Learned Potential for Krypton under Extreme Conditions
Source: J Phys Chem Lett. 2025 Feb 4;16(6):1559–66. doi: 10.1021/acs.jpclett.4c03272 (PMC11831645; doi:10.1021/acs.jpclett.4c03272)
Supplement: Supplementary file 1 — jz4c03272_si_001.pdf [file jz4c03272_si_001.pdf]

jz-2024-03272y.R1

Name: Peer Review Information for "An Accurate Machine-Learned Potential for Krypton at Extreme Conditions"

First Round of Reviewer Comments

Reviewer: 1

Comments to the Author

This is in principle a nice paper for JPCL. It introduces the reader nicely into what can be currently done in this field. However, some references of previous work are clearly missing and some additional comments are included below. After these has been considered, the paper can go into production.

Introduction:

May I make the authors aware of a review on Lennard Jones by their colleague at Cambridge,

“This is especially true for fluid states where many-body terms are widely thought of as having less significance than in solids and so a pairwise model does well to provide a good approximation of the interactions.” It has indeed less significance but I do not think that a many-body expansion of the interaction potential terminating the expansion at a low finite order will work for metallic fluids. That is why other potentials such as the glue potential are used.

“Similarly, the attractive r−6 term increases more rapidly out of the potential minimum, leading to weaker attractive interactions between particles. Reference needed.” There are many other terms in the perturbation treatment of weakly interacting systems, but the dominant one at long range is r^{-6}. So be more clear here.

Computational section:

Careful overstating the accuracy of CCSD(T). Specially the triples are done perturbatively which at short distance may not be accurate. At long distance one gets away with having closed shell atoms, otherwise the perturbative triples are known to lead to divergencies. Check the literature.

What is the advantage of using blip functions instead of for example splines of Chebyshev polynomials?

What about the angle dependence of the 3-body term? As I can see it is not included unlike the case of the Axilrod-Teller-Muto potential.

The different algorithms for melting simulations are discussed for example in Comput. Mat. Sci. 124 (2016) 335–343.

I think there is still a lack for proving why the Z method seems to compensate for superheating, but I guess this is covered by stating that it is empirically observed.

DFT does not correctly describe many-body interactions and for Krypton one needs to include dispersion forces which can be done by using Grimme's correction.

I believe the solid-liquid phase coexistence method used here is also known as interface pinning, right?

Results: Considering krypton melting there is also a paper out on this, see Phys. Rev. B 101, 104103-1-12 (2020). These authors discuss in detail 3-body effects and overheating.

I believe for the (12,6)-Lennard-Jones potential the melting point has been accurately determined, see for example J. Chem. Phys. 127, 104504 (2007).

For high pressure melting and the deviations to experiment see for example Angew. Chem. Int. Ed. 52, 13202-13205 (2013).

Talking about experiment, what about the work by Phys. Rev. Lett. 86, 5731 (2001) and Phys. Rev. B 57, 230 (1998).

Reviewer: 2

Comments to the Author

The paper reports the development of new machine learned potentials for Krypton based on CCSD(T) calculations on dimers and trimers. Both potentials were found to give good agreement with the experimental equation of state, melting point, and neutron scattering data for the fluid. The new potentials produced similar results to the widely used Lennard-Jones potential for low-pressure melting and equation of state. For higher pressures up to 30 GPa the existing Lennard-Jones showed a notable divergence from the experimental (solid) equation of state while the new potentials showed extremely good agreement. This is despite not having any solid phase data in their training set.

The paper is significant, and it will be of interest to the readership of the Journal of Physical Chemistry Letters. I suggest a revision in which the authors consider addressing the following comments:

1) p. 4, line 5: the superscript 12 and 6 should be rather referred as 1/r^12 and 1/r^6 as in the rest of the manuscript

2) p. 5: give more information about the 36 data from Ref. 10 used to fit the 2-body potential, i.e. basis set, possible inclusion of BSSE etc.

3) regarding the 3-body corrections: a) how many trimer points at equilateral geometries were used? b) were they derived at the same level as the 36 points for the 2-body? c) are these new data or they were also taken from Reference 10?

4) a general comment: nowadays the use of just 36 2-body points is outdated – many more can be computed especially in the repulsive and long range regions of the potential

5) I wonder what the magnitude of the 3-body terms is compared to the 2-body ones

6) in eq. (3) perhaps the e_i should be rather replaced with e_ij

7) the authors compare the results with the LJ potential of Ratkai et al. (ref 6). I wonder how the older potential developed by Aziz performs

8) the authors may want to consider for this or future studies to use the generalized LJ potential described in JCP vol. 141, 064117 (2014) and JCP vol. 141, 064118 (2014) as it has an additional flexibility that describes both the short range repulsive and long range attractive parts of the potential better than the LJ using just one additional parameter than the LJ.

6) finally, I wonder whether this paper meets the length requirements for JPC Letters, it seems too long.

The paper can be accepted after the authors address the above comments.

Author's Response to Peer Review Comments:

Centre for Science at Extreme Conditions
Institute for Condensed Matter and Complex Systems
School of Physics and Astronomy
University of Edinburgh
James Clerk Maxwell Building
Edinburgh
EH9 3FD
gjackland@ed.ac.uk

Corresponding author:

Graeme J Ackland

Senior Editor
The Journal of Physical Chemistry Letters

19th December 2024

Dear Editor,

We are pleased to resubmit the manuscript titled ‘An Accurate Machine-Learned Potential for Krypton at Extreme Conditions’ to the Journal of Physical Chemistry Letters, specifically to the ‘Physical Insights into Materials and Molecular Properties’ section.

We would like to thank the reviewers for their careful reading of the manuscript and thoughtful comments. We offer below a detailed answer to the issues raised and highlight the changes made to the manuscript to better convey our intended message and strengthen the arguments in favour of our conclusions.

In what follows, the reviewers’ comments are coloured in dark blue and our replies are coloured in black.

“**Reviewer #1**

Recommendation: This paper is probably publishable, but major revision is needed; I do not need to see future revisions.

Comments:
This is in principle a nice paper for JPCL. It introduces the reader nicely into what can be currently done in this field. However, some references of previous work are clearly missing and some additional comments are included below. After these has been considered, the paper can go into production.

We thank the reviewer for their appreciation and the very constructive comments to which we respond below!

Introduction:
May I make the authors aware of a review on Lennard Jones by their colleague at Cambridge,

“This is especially true for fluid states where many-body terms are widely thought of as having less significance than in solids and so a pairwise model does well to provide a good approximation of the interactions.” It has indeed less significance but I do not think that a many-body expansion of the interaction potential terminating the expansion at a low finite order will work for metallic fluids. That is why other potentials such as the glue potential are used.

We thank the reviewer for pointing out this omission on our behalf, and have now amended the text accordingly to clearly state such well-known limitations, and guided the reader to the very thorough review by Schwerdtfeger and Wales.

“Similarly, the attractive r−6 term increases more rapidly out of the potential minimum, leading to weaker attractive interactions between particles. Reference needed.” There are many other terms in the perturbation treatment of weakly interacting systems, but the dominant one at long range is r^{-6}. So be more clear here.

We have clarified the statement above to make it explicit that we are comparing to empirical potentials capable of representing the real diffraction data as obtained in a previous study, which we now also reference in place.

Computational section:

Careful overstating the accuracy of CCSD(T). Specially the triples are done perturbatively which at short distance may not be accurate. At long distance one gets away with having closed shell atoms, otherwise the perturbative triples are known to lead to divergencies. Check the literature.

The perturbative approach to triples may introduce inaccuracies at short distances due to stronger and more complex interactions. However, it is hard to quantify how this will translate to bulk properties. Based on the success of our model, in particular at high density compared with Rutkai-LJ, it appears that for Krypton these effects are unimportant. This may be due to the fact that we have closed shell configurations, and lower polarisability than in heavier noble gas molecules. It is an important, and surprising, result from our work that these triplet terms are unimportant: the most significant failing of previous work is the overly hard short ranged repulsion.

What is the advantage of using blip functions instead of for example splines of Chebyshev polynomials?

BLIP functions have local (compact) support, meaning changes to one part of the function do not affect the entire domain. Fundamentally, BLIPs are made of B-splines to imitate Gaussians but with compact support. This also results in increased computational efficiency. They provide better smoothness and stability compared to Chebyshev polynomials, which can suffer from oscillations.

What about the angle dependence of the 3-body term? As I can see it is not included unlike the case of the Axilrod-Teller-Muto potential.

We have now added some clarification and stated explicitly in the manuscript that while our potentials may include 3 and 3-body energy contributions from the CCSD(T) calculations, the potentials themselves are simple pair potentials, and hence do not have a 3-body angular term that is computed.

The different algorithms for melting simulations are discussed for example in Comput. Mat. Sci. 124 (2016) 335–343.

We thank the reviewer for pointing this article out to us. We have now included for completeness a reference in text pointing the reader to both this and another in-depth review of melting simulations.

I think there is still a lack for proving why the Z method seems to compensate for superheating, but I guess this is covered by stating that it is empirically observed.

We agree with the reviewer on the lack of clarity still surrounding the Z method. This is indeed the reason we stipulated that it is empirically observed to work well and added a number of references to existing literature discussing the method and its rationale in greater detail than in our current work.

DFT does not correctly describe many-body interactions and for Krypton one needs to include dispersion forces which can be done by using Grimme's correction.

This is a correct statement, and precisely why we used CCSDT rather than DFT as our training set.

I believe the solid-liquid phase coexistence method used here is also known as interface pinning, right?

The method we use here is slightly different from interface pinning, as there is no bias potential used to maintain the existence of the two phases during the course of the simulation. We have now added an explicit statement to this effect to avoid any potential confusion.

Results:

Considering krypton melting there is also a paper out on this, see Phys. Rev. B 101, 1041031-12 (2020). These authors discuss in detail 3-body effects and overheating.

We thank the reviewer for pointing us to this study. We have now included it and stressed in the text the liability of krypton to superheating and the effect of 3-body interactions in manybody potentials for it.

I believe for the (12,6)-Lennard-Jones potential the melting point has been accurately determined, see for example J. Chem. Phys. 127, 104504 (2007).

We thank the reviewer for their suggestion, which we have cited now in our manuscript and commented on its relevance as well as good agreement with our current results in the relevant p-T range.

For high pressure melting and the deviations to experiment see for example Angew. Chem. Int. Ed. 52, 13202-13205 (2013).

Talking about experiment, what about the work by Phys. Rev. Lett. 86, 5731 (2001) and Phys. Rev. B 57, 230 (1998).

We thank the reviewer for pointing out these items of previous research to us. We have now ammended the text, mentioning these works and contextualising them appropriately with respect to our current aims and results.

Additional Questions: Urgency: High

Significance: Top 10%

Novelty: High

Scholarly Presentation: High

Is the paper likely to interest a substantial number of physical chemists, not just specialists working in the authors' area of research?: Yes

Reviewer #2

Recommendation: This paper may be publishable, but major revision is needed; I would like to be invited to review any future revision.

Comments:
The paper reports the development of new machine learned potentials for Krypton based on CCSD(T) calculations on dimers and trimers. Both potentials were found to give good agreement with the experimental equation of state, melting point, and neutron scattering data for the fluid. The new potentials produced similar results to the widely used Lennard-Jones potential for low-pressure melting and equation of state. For higher pressures up to 30 GPa the existing Lennard-Jones showed a notable divergence from the experimental (solid) equation of state while the new potentials showed extremely good agreement. This is despite not having any solid phase data in their training set.

We thank the reviewer for their careful reading of our manuscript and for their appreciation.

The paper is significant, and it will be of interest to the readership of the Journal of Physical Chemistry Letters. I suggest a revision in which the authors consider addressing the following comments:

1) p. 4, line 5: the superscript 12 and 6 should be rather referred as 1/r^12 and 1/r^6 as in therest of the manuscript

We have amended the mentioned line to ensure consistency with the rest of the manuscript.

2) p. 5: give more information about the 36 data from Ref. 10 used to fit the 2-body potential,i.e. basis set, possible inclusion of BSSE etc.
3) regarding the 3-body corrections: a) how many trimer points at equilateral geometries wereused? b) were they derived at the same level as the 36 points for the 2-body? c) are these new data or they were also taken from Reference 10?
4) a general comment: nowadays the use of just 36 2-body points is outdated – many more can be computed especially in the repulsive and long range regions of the potential 5) I wonder what the magnitude of the 3-body terms is compared to the 2-body ones

We expanded our description of the source data from Ref. 10 in the manuscript to include more information about the basis set and the number and span of the 3-body correction points, which were also taken from Ref. 10 for consistency. For an interested reader, the full details of the CCSD(T) calculations can be found in the original article (ref. 10).

6) in eq. (3) perhaps the e_i should be rather replaced with e_ij

We thank the referee for pointing out the confusing explanation we provided for the equation. We have now amended the text to more explicitly try to avoid such confusion, bearing in mind this is still a rather technical and abstract aspect of machine-learning and energy decomposition. In our case, e_i represents local atomic energies which are computed by summing over bond energies e_{ij} (eq. 4).

7) the authors compare the results with the LJ potential of Ratkai et al. (ref 6). I wonder howthe older potential developed by Aziz performs
8) the authors may want to consider for this or future studies to use the generalized LJpotential described in JCP vol. 141, 064117 (2014) and JCP vol. 141, 064118 (2014) as it has an additional flexibility that describes both the short range repulsive and long range attractive parts of the potential better than the LJ using just one additional parameter than the LJ.

From our experience, we found that significantly different functional forms (energy curves) can sometimes yield very similar observables. Conversely, minor changes in energy curvature can result in surprisingly large differences. A fair comparison would require computing many physical quantities, which is beyond the scope of this work. More importantly, the potential of Rutkai et al. was used in the Reverse Monte Carlo (RMC) fitting of the experimental diffraction data that we compare the liquid structure to. Consequently, the exact ways in which it comes short of representing real fluid krypton from a structural point of view are known. Unfortunately, RMC fitting with other types of potentials (such as Aziz, generalised LJ, etc.) is a currently unavailable feature. Nevertheless, we thank the reviewer for their suggestions and as a consequence have mentioned in the text the generalised potential and its improvements over the canonical version. We believe this addition makes for a more complete contextualisation of our current findings within modern computational chemistry.

6) finally, I wonder whether this paper meets the length requirements for JPC Letters, it seems too long.

We ran our manuscript through a latex word counter and believe that it is within the length requirements of the J. Phys. Chem. Lett. guidance.

The paper can be accepted after the authors address the above comments.

Additional Questions: Urgency: Moderate

Significance: High

Novelty: Moderate

Scholarly Presentation: High

Is the paper likely to interest a substantial number of physical chemists, not just specialists working in the authors' area of research?: Yes

End response

We hope our response addresses thoroughly the issues raised by the reviewers and would once again like to thank them for their constructive comments and recommendations!

It is our hope that the manuscript is now in an appropriate shape to be published in your journal!

Yours sincerely, Ciprian G.

Pruteanu

Postdoctoral Research Associate
School of Physics and Astronomy
University of Edinburgh
James Clerk Maxwell Building
Peter Guthrie Tait Road
Edinburgh, United Kingdom
EH9 3FD
Email: cip.pruteanu@ed.ac.uk

Graeme J. Ackland
Professor of Computer Simulation
School of Physics and Astronomy
University of Edinburgh
James Clerk Maxwell Building
Peter Guthrie Tait Road
Edinburgh
EH9 3FD, United Kingdom
Email: gjackland@ed.ac.uk

jz-2024-03272y.R2

Name: Peer Review Information for "An Accurate Machine-Learned Potential for Krypton at Extreme Conditions"

Second Round of Reviewer Comments

Reviewer: 2

Comments to the Author

In the revised manuscript the authors have addressed most of the comments while leaving a few unanswered. For instance, they did not address comment #5 of reviewer #2 (relative magnitude of 3- vs. 2-body terms). They added the text on p. 6: "This is

based on the assumption that the three-body corrections will be negligibly small at higher

separation distances," They have both the 2- and 3-body terms at shorter distances and they can see what the ratio of their values is and how it varies with intermolecular separation to check whether this statement is correct.

As regards comment #7 about the Aziz potential, I was not asking to perform new simulations with that old potential but they could have added the comparison of that interatomic potential in Figure 2 or in the SI.

Author's Response to Peer Review Comments:

.

Centre for Science at Extreme Conditions
Institute for Condensed Matter and Complex Systems
School of Physics and Astronomy
University of Edinburgh
James Clerk Maxwell Building
Edinburgh
EH9 3FD
cip.pruteanu@ed.ac.uk

Corresponding author:

Ciprian G. Pruteanu
Postdoctoral Research Fellow

Senior Editor
The Journal of Physical Chemistry Letters

26th December 2024

Dear Editor,

We are pleased to resubmit the manuscript titled ‘An Accurate Machine-Learned Potential for Krypton at Extreme Conditions’ to the Journal of Physical Chemistry Letters, specifically to the ‘Physical Insights into Materials and Molecular Properties’ section.

We would like to thank the reviewers for their careful second reading of the manuscript and final comments. We offer below a detailed answer to the latest minor issues raised and highlight the changes made to the manuscript to better convey our intended message and strengthen the arguments in favour of our conclusions.

We have also implemented all the formatting changes requested by the editorial office.

In what follows, the reviewers’ comments are coloured in dark blue and our replies are coloured in black.

“**Reviewer #2**

Recommendation: This paper is publishable subject to minor revisions noted. Further review is not needed.

Comments:
In the revised manuscript the authors have addressed most of the comments while leaving a few unanswered. For instance, they did not address comment #5 of reviewer #2 (relative magnitude of 3- vs. 2-body terms). They added the text on p. 6: "This is based on the assumption that the three-body corrections will be negligibly small at higher separation distances," They have both the 2- and 3-body terms at shorter distances and they can see what the ratio of their values is and how it varies with intermolecular separation to check whether this statement is correct.

We have now added a statement stating exactly the relative magnitude of the 2-body and 3-body energy terms and their evolution with increasing atom-atom separation, and have also indicated explicitly how this can be seen and confirmed by the total energy curves depicted in Figure 2.

As regards comment #7 about the Aziz potential, I was not asking to perform new simulations with that old potential but they could have added the comparison of that interatomic potential in Figure 2 or in the SI.

We have now added the Aziz potential into figure 2 and the graphical abstract, and increased the text size as requested.

The first author has also changed her surname.

Additional Questions:

Urgency: Moderate

Significance: High

Novelty: Moderate

Scholarly Presentation: High

Is the paper likely to interest a substantial number of physical chemists, not just specialists working in the authors' area of research?: Yes

End response

We hope our response addresses thoroughly the issues raised by the reviewers and would once again like to thank them for their constructive comments and recommendations!

It is our hope that the manuscript is now in an appropriate shape to be published in your journal!

Yours sincerely, Ciprian G.

Pruteanu

Postdoctoral Research Associate
School of Physics and Astronomy
University of Edinburgh
James Clerk Maxwell Building
Peter Guthrie Tait Road
Edinburgh, United Kingdom
EH9 3FD
Email: cip.pruteanu@ed.ac.uk

Graeme J. Ackland
Professor of Computer Simulation
School of Physics and Astronomy
University of Edinburgh
James Clerk Maxwell Building
Peter Guthrie Tait Road
Edinburgh
EH9 3FD, United Kingdom
Email: gjackland@ed.ac.uk
